# Function of Dicer with regard to Energy Homeostasis Regulation, Structural Modification, and Cellular Distribution

**DOI:** 10.1155/2020/6420816

**Published:** 2020-07-25

**Authors:** Xiaohui Tong, Nianjun Yu, Rongchun Han, Tongsheng Wang

**Affiliations:** ^1^School of Life Sciences, Anhui University of Chinese Medicine, Hefei, China; ^2^School of Pharmacy, Anhui University of Chinese Medicine, Hefei, China

## Abstract

As a type III ribonuclease (RNase III) specifically cleaving double-stranded RNA substrates into short fragments, Dicer is indispensable in a range of physi/pathologic processes, e.g., nutrient deprivation, hypoxia, or DNA damage. Therefore, much interest has been paid to the research of this protein as well as its products like microRNAs (miRNAs). The close relationship between Dicer levels and fluctuations of nutrient availability suggests that the protein participates in the regulation of systemic energy homeostasis. Through miRNAs, Dicer regulates the hypothalamic melanocortin-4 system and central autophagy promoting energy expenditure. Moreover, by influencing canonical energy sensors like adenosine monophosphate-activated protein kinase (AMPK) or mammalian target of rapamycin (mTOR), Dicer favors catabolism in the periphery. Taken together, Dicer might be targeted in the control of energy dysregulation. However, factors affecting its RNase activity should be noted. Firstly, modulation of structural integrity affects its role as a ribonuclease. Secondly, although previously known as a cytosolic endoribonuclease, evidence suggests Dicer can relocalize into the nucleus where it could also produce small RNAs. In this review, we probe into involvement of Dicer in energy homeostasis as well as its structural integrity or cellular distribution which affects its ability to produce miRNAs, in the hope of providing novel insights into its mechanism of action for future application.

## 1. Introduction

Dicer was identified in 2001 as a member of RNase III family enzyme that specifically cleaves long dsRNA substrates into short dsRNA fragments of defined length, typically around 21–25 nucleotides [1]. This evolutionarily conserved and universally expressed protein [1, 2] plays crucial physiological roles [3] and its absence is associated with various disease development stages [4, 5]. In recent years, the type III RNase Dicer has emerged as a key regulator of cellular adaptive response to fluctuation of nutrient availability as well as metabolic homeostasis. For example, mRNA levels of miRNA maturation machinery—Drosha, Dgcr8, Dicer, and Argonaute-2—are significantly increased *in vitro* in the human umbilical vein endothelial cells (HUVECs) by hyperglycemia, indicating that Dicer or miRNAs are sensitive to nutrient alterations [6]. In addition, sulfur amino acid restriction (i.e., methionine or cysteine) is sufficient to increase Dicer mRNA level in preadipocytes, similar to the effect of increased Dicer expression in adipose tissue induced by dietary restriction [7]. Moreover, the result that Dicer and miRNAs decrease gradually in adipose tissue during aging, which is associated with age-related insulin resistance [8], further supports the notion that Dicer might mediate beneficial metabolic effects in balancing systemic energy.

Systemic energy homeostasis is finely controlled by various balance regulation systems, one of which is the hypothalamic melanocortin system. Proopiomelanocortin (POMC) neurons and neurons coexpressing agouti-related protein (AgRP) and neuropeptide Y (NPY) in the arcuate nucleus of the hypothalamus (ARH) are important components in this system. POMC neurons function to reduce food intake and increase energy expenditure by releasing *α*-melanocyte-stimulating hormone (*α*MSH) that activates melanocortin-4 receptors (MC4R), whereas AgRP acts as an endogenous antagonist of MC4R [9]. Through miRNAs-dependent mechanisms, central Dicer is required for systemic fat expenditure and glucose consumption via the hypothalamic melanocrotin-4 system [10, 11].

Autophagy is a cellular catabolic process that delivers cytoplasm constituents such as macromolecules or subjects damaged organelles to lysosome for degradation to maintain energy homeostasis and to fight against cellular stress. Interestingly, the convergence of Dicer and autophagy in response to multiple forms of cellular stresses, such as nutrient deprivation [12], hypoxia [13, 14], DNA damage [15], and heat shock [16], suggests that Dicer might have an important role in aiding cellular survival through participation in catabolic metabolism in adversity [17].

The integration of nutrient status and metabolic homeostasis is a complex process in multicellular organisms, and the responsiveness of an organism to nutritional stress is critical for its survival. Therefore, do these results suggest that Dicer could be targeted in energy dysregulation? Current literature indicates Dicer is an enzyme of extraordinary versatility. To some extent, function of Dicer depends on its structural integrity and modulation of Dicer domains might jeopardise its role as a ribonuclease [18]. In addition, although previously seen as a cytosolic endoribonuclease, emerging evidence supports that Dicer is also detectable in the nucleus [19]. So this versatility suggests extra thoughts should be taken into consideration when looking for specific pharmacological compounds targeting Dicer. In this study, we review the role of Dicer in energy homeostasis in terms of its central and peripheral influence, as well as the potential of Dicer as a therapeutic target in improving dysregulation of energy homeostasis.

## 2. Regulation of Catabolism by Dicer in the Central Nervous System

The central nervous system (CNS) monitors modifications of metabolic parameters or hormone levels and elicits adaptive responses like modulation of food intake or autonomic nervous system. Various studies on central Dicer knockout (KO) phenotype reveal that central Dicer is implicated in the regulation of energy expenditure. Mutation of Dicer in neurons of adult mice causes chronic activation of the signaling of phosphoinositide 3-kinase (PI3K)/protein kinase B (Akt)/mTOR due to loss of mir-103, resulting in severe hyperphagic obesity [20]. Specifically, Dicer KO in hypothalamic POMC neurons induces obesity and hyperglycemia caused by hyperphagia in combination with declined energy expenditure [10, 11]. Moreover, aging witnesses a decline in Dicer level in the hypothalamus, leading to differentiation of *Pomc*-expressing progenitors into AgRP/NPY phenotype and subsequent metabolic dysregulation, which is possibly mediated by mir-107/103 [21]. Thus, it is assumable that, through specific miRNAs like mir-103 in POMC neurons, Dicer positively promotes catabolic metabolism. Indeed, monoallelic depletion of Dicer in POMC neurons incurs reduced metabolic rates in mice by reduction of O_2_ consumption and CO_2_ production. However, food intake is decreased due to elevated leptin sensitivity in POMC neurons demonstrated by increased phosphorylation of signal transducer and activator of transcription 3 (STAT3) in POMC neurons upon leptin administration [22]. Leptin, an adipokine secreted by adipocytes, acts on POMC and AgRP/NPY neurons to suppress food intake and promote energy expenditure [23]. The discrepancy that, by manipulation of Dicer, food intake does not coincide [10, 11, 22] might be explained by the fact that partial Dicer defect predominantly affects metabolic rates. And as a compensator, leptin sensitivity is subsequently increased to maintain normal body weight. However, this assumption needs further confirmation.

Autophagy can respond to cellular stresses including hypoxia, growth factor, or nutrient deprivation [24]. During starvation, cytoplasmic materials are degraded by autophagy to yield fatty acids and amino acids which are then utilized to produce new proteins or to synthesize ATP for cell survival [25]. Dicer might also affect metabolism through autophagy. By using GFP-LC3 mice, Alirezaei et al. proved that autophagy is dramatically induced in cortical neurons and Purkinje cells by short-term fasting, demonstrated by changes in autophagosome abundance and diminished neuronal mTOR *in vivo* [26]. Interestingly, Dicer is demonstrated to be a prerequisite for the induction of autophagy caused by nutrient deprivation in primary cortical neurons *in vivo*, partly via Let-7 production which subsequently activates autophagy through inhibition of mTOR activity [27].

Therefore, central Dicer positively regulates the hypothalamic melanocortin-4 system and autophagy process in the CNS mediated by related miRNAs.

## 3. Regulation of Catabolism by Dicer in the Periphery

In the periphery, a series of Dicer KO studies indicate that Dicer deficiency results in excess energy deposition. For instance, tamoxifen-inducible KO of Dicer in around eight-week-old mice leads to severe lipid accumulation in small intestinal epithelium, associated with abnormal expressions of enzymes involved in lipid metabolism, including microsomal triglyceride transfer protein (MTTP), dihydroxyacetone kinase (DAK), and very long chain fatty acyl-CoA dehydrogenase (VLCAD) [28]. Researches of conditional Dicer KO phenotype confirm that Dicer is required for energy metabolism in different tissues and cells. A case in point is that hepatic Dicer depletion is associated with lipid and glycogen accumulation in liver, linked with increased cell proliferation and risks of carcinomas [29]. In addition, studies of Dicer KO in adipose tissue, a major player in glycemic control and nutrient homeostasis, demonstrate metabolic defects such as lipodystrophy, inflammation, or dyslipidemia, accompanied by insulin resistance [30], which might also be associated with structural and functional dysfunctions in mitochondria [31]. Mitochondria control many biological processes, most importantly aerobic metabolism to generate ATP, a process known as oxidative phosphorylation (OXPHOS). Interestingly, Dicer is a positive regulator of fatty acid oxidation and OXPHOS in macrophages. *In vivo*, conditional Dicer deletion in macrophages in mice accelerates atherosclerosis caused by lipid accumulation via disruption of fatty acid-fueled mitochondrial respiration [32]. Also, in cardiac mesenchymal stem cell (C-MSC), downregulation of Dicer modulates cellular metabolism to accommodate diminished oxygen and substrate supply associated with heart failure, by reducing mitochondrial respiration and decreasing the expression of related enzymes involved in fatty acid *β*-oxidation [33].

Therefore, in the periphery, Dicer also seems to act as a promoter for energy expenditure. Evidence shows that classical energy sensors like AMPK, which are induced by increased ratio of cellular AMP to ATP, affect Dicer activity. For example, Dicer can be upregulated by AMPK activator metformin which produces mir-33a to target c-MYC, insulin receptor substrate 2 (IRS2), and hypoxia-inducible factor alpha (HIF*α*) to switch glucose from nucleotide biosynthesis to catabolic pathways and subsequently reduce the incidence of cancer and cancer-related mortality in diabetic patients [34]. Interestingly, KO of TAp63, a member of p53 family [35], displays defects in fatty acid oxidation and manifests obesity in mice due to inhibition of metformin responsive genes like AMPK, Sirt1, and LKB1 [36]. Since chromatin immunoprecipitation (ChIP) analysis shows that all isoforms of TAp63 (*α*, *β* and *γ*) are able to regulate *Dicer* transcriptionally [37], it would be interesting to investigate whether Dicer is downstream of TAp63 or upstream of AMPK for energy catabolism.

Intriguingly, in the fat body of *Drosophila melanogaster* (counterpart of mammalian adipose tissue and liver), starvation negatively affects miRNA producing machinery including Dicer, alleviating mir-305 dependent targeting of the *dp53-3*′ untranslated region (UTR) and contributing to reduced sugar consumption and compromised resistance to energy deprivation [12]. But in what way Dicer is inhibited by starvation is unknown. Later researches suggest that autophagy might have a role in the regulation of Dicer by nutrient depletion. Indeed, autophagy activation by serum starvation or mTOR inhibitor rapamycin decreases Dicer and Argonaute-2 levels, whereas autophagy blockade by inhibitors of lysosomal acidification (bafilomycin A1 or chloroquine) increases their levels in a broad range of cancer cell lines like HeLa cells, MDA-231, T47D, and MDA-435. Autophagy can influence Dicer expression at two distinct levels: through NDP52-dependent protein degradation and through post‐transcriptional control of Dicer transcripts by Let-7a [38]. Activation of mTOR by targeted mutation of *Tsc1*, a negative regulator of mTORc1, causes a mild increase in Dicer protein level [39]. Meanwhile Dicer ubiquitination by the E3 ligase Parkin under hypoxia also enhances its degradation via autophagy in multiple types of cancer cell lines and human tumors, the consequence of which is the loss of several tumor suppressor miRNAs [40]. Surprisingly, Dicer can also modulate autophagy in turn. For instance, silencing of Dicer1 in MEC-1 and primary chronic lymphocytic leukemia (CLL) cells leads to reduced autophagic flux by which several cancer cells exploit to overcome stress like starvation and hypoxia [41]. Similarly, Dicer inhibition attenuates the activation of autophagy that is needed for proper neutrophil differentiation of AML cells [42] and the possible explanation is its capability to produce antiautophagy miRNAs. For example, deprivation of Dicer-derived mir-124 and mir-144 by hypoxia contributes to the loss of inhibition of PIM1, a well-recognized oncogene of prostate cancer [43] and subsequent activation of autophagy in prostate cancer cells (DU145 and PC3 cells) [44]. Additionally, mir-26b targets the 3′ UTR of unc-51-like autophagy activating kinase 2 (ULK2), a serine/threonine protein kinase that affects mTOR and ATG related progression of the catabolic process in autophagy [45].

**Figure 1:**
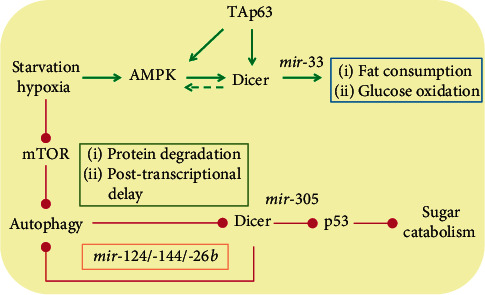
Catabolic roles of Dicer in a miRNAs-dependent manner in the periphery.

Currently, the reasons leading to the contradictory alterations of Dicer level or activities by nutrient depletion are not well exploited [10, 11, 22]. This may be due to different experimental contexts or models and awaits further confirmation. Collectively, the results suggest a catabolic role of peripheral Dicer via production of distinct miRNAs in the regulation of basal energy homeostasis as well as in response to nutrient starvation (summarized in Figure 1). Thus, is it possible to develop pharmacological compounds to target the RNase Dicer in order to treat energy dysregulation? To answer this question, several aspects affecting the RNase activity of Dicer should be noted.

## 4. Structural Modification Affects RNase Activity of Dicer

**Figure 2:**
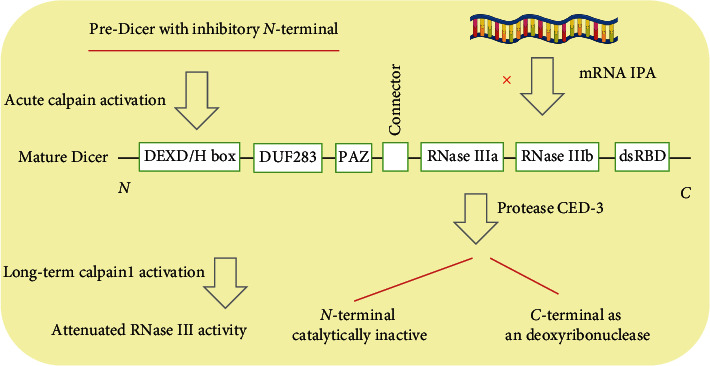
Schematic representation of Dicer structural modulations.

Emerging evidence supports that RNase activity of Dicer is associated with its structural integrity and is subjected to modifications in different contexts (summarized in Figure 2). Structurally, in addition to two tandemly arranged RNase III domains (RIII Da and RIII Db) and a double-strand RNA-binding domain (dsRBD) in the carboxyl terminus, Dicer protein usually has an amino-terminal DEXD/H-box domain, followed by a small domain of unknown function (DUF283) and a Piwi/Argonaute/Zwilli (PAZ) domain [46]. Maturation of its structure depends on some proteases. For example, Dicer is enriched but enzymatically inactive in postsynaptic densities (PSDs), which would be liberated in active form with RNase III activity by Calpain cleavage [47]. However, Elgheznawy et al. report that the proteolysis of Dicer by long term activation of Calpain1 is blamed to be responsible for the markedly attenuated miRNA levels and function in platelet in patients with diabetes mellitus, with the treatment of Calpain1 inhibitor A-705253 rescuing this alteration [48]. But the exact proteolytic site in Dicer by long term Calpain1 exposure in platelet is unsolved. Moreover, although Dicer is gradually lost in many tissues during aging in mammals [49–51], the level of Dcr-1 (homology of mammalian Dicer1) is negatively associated with embryonic survival rates in *Caenorhabditis elegans* [52]. Then why does this happen? It is found that phosphorylation of *C. elegans* Dcr-1 in the RNase IIIb and dsRNA-binding domain mediated by ERK alters Dcr-1 activity [52]. Therefore, the sheer quantity does not reflect the capability of Dcr-1 comprehensively. Another case is that CED-3 caspase cleavages Dcr-1 at aspartate^1472^, which is in the middle of RNase IIIa domain, abolishing its 22- to 23-nucleotide dicing activity. However, the C-terminal of Dcr-1 still contains one intact RNase III domain that may retain some endonuclease activity, producing *3*′ *hydroxyl* DNA breaks on chromosomes and promoting apoptosis. Therefore, Dcr-1 undergoes a protease-mediated conversion from a ribonuclease to a deoxyribonuclease by the CED-3 cleavage during apoptosis [18]. Pathological modifications of Dicer structure also occur to mammalian cells. In malignant B cells from patients with CLL, the RNA sequencing or 3′-end sequencing technique detects a widespread upregulation of truncated Dicer mRNAs caused by intronic polyadenylation (IPA), leading to abundant truncated Dicer protein. In contrast with its full-length structure, IPA of Dicer entirely lacks miRNA cleavage ability and mimics truncating mutations with both RNase III domains removed, therefore losing its role as a tumor suppressor [53].

## 5. Cellular Distribution Affects Dicer Function

Regarding cellular distribution of Dicer, data from current literature seem controversial. Previously, Dicer was believed to be a cytoplasmic protein. Mammalian cells such as CHO, Cos-7, or HeLa cells, transiently transfected with constructs encoding MYC-tagged human Dicer for 18–24 h, exhibit exclusive cytoplasmic staining of Dicer with no nuclear signal detected [54]. Immunohistochemistry (IHC) using the C-terminal anti-Dicer antibody with fluorescence detection reveals that Dicer is expressed in many brain regions in a punctate pattern in the somatodendritic compartment of large neurons, some interneurons, and endothelial cells in mice. Furthermore, electron microscopy detection confirms that Dicer is present within dendritic spines and appears to be particularly associated with PSDs. Surprisingly, in cortical pyramid neurons, intense streaks of Dicer immunoreactivity are commonly found within a subset of nuclei [47]. So, is Dicer also located in the nucleus?

Dicer is associated with transcribed regions of both active and silenced genes in ribosomal DNA (rDNA) chromatin repeats regardless of their transcriptional activity during mitosis in vertebrate cultured cells, although the role is not readily apparent [55]. The group of David R. Corey reports the presence of RNA interference factors: Argonaute-2, Dicer, TRBP, and TRNC6A/GW182 in the nucleus of HeLa cells. Interestingly, sequencing of small RNAs reveals that, out of 456 miRNA species identified in the whole cell, 346 of them also exist in the nucleus, suggesting that roughly 75% of the miRNAs in the cytoplasm are shuttled into the cell nucleus. In addition, many of the top enriched miRNAs are shared between nuclei and whole cells, indicating a similar distribution of abundant miRNA species in the cytoplasm and the nucleus [19, 56]. Interestingly, the isolated nuclear Dicer is enzymatically active to produce mature miRNAs *in vitro* [19].

Furthermore, in fission yeast *Schizosaccharomyces pombe*, Dcr-1 is reported to be a bona fide nuclear protein that associates with nuclear pore complexes (NPCs) via a 33-amino-acid extension in the C terminus (C33), with deletion of C33 in Dcr-1 resulting in dramatic loss of nuclear Dicer. dsRBD domain of Dcr-1 also mediates nucleocytoplasmic trafficking. Thus, it is assumable that Dicer might be a protein shuttling among subcellular compartments [57, 58]. Indeed, nonstressfully, in *S. pombe*, Dcr-1 is located at nuclear pores for repression of centromeric heterochromatin, but would translocate into cytoplasm by stressors like heat shock [16]. So does this also happen in mammalian cells? In cholangiocarcinoma (CCA) cells, Dicer translocates into the nucleus to form a complex with heterochromatin protein 1*α* (HP1*α*), contributing to tumorigenesis. This nuclear Dicer/HP1*α* complex functions to promote the DNA methylation and inhibition of the promoter of secreted frizzled-related protein 1 (SFRP1), which is an antiproliferative tumor suppressor [59]. Additionally, Dicer is recruited to chromatin in a ZRF1-dependent manner in response to ultraviolet irradiation in HEK293T, U2OS, and U2OS 2-6-3 cells. Then, the H2A-ubiquitin binding protein ZRF1 and Dicer together mediate the decondensation of chromatin [60], which is a prerequisite for removing helix distorting DNA lesions such as 6-4 photoproducts and cyclobutane pyrimidine dimmers from chromatin [61]. Controversially, Burger et al. have reported that Dicer expression is not significantly affected in HEK293 cells after continuous exposure with DNA damaging inducing agents including etoposide, hydrogen peroxide, phleomycin, methyl methanesulfonate, or *γ*-irradiation. Rather, in response to DNA damage, Dicer nuclear accumulation is promoted via phosphorylation of residue S1016 in the platform-PAZ-connector helix [62]. Nuclear Dicer, phosphorylated at residues S1016 and S1728/S1825, promotes recruitments of DNA repair factors MDC1 and 53BP1 to DNA double-strand breaks (DSBs) [62]. However, in another study from this team, intrinsic immunoblots fail to detect the nuclear phosphorylated HA-Dicer previously shown by their immunofluorescence (IF), and they consider that might be because the p-Dcr-1 antibodies are primarily suited for IF microscopy but have limited performance in immunoblotting [63]. Nevertheless, Drake et al. show that, in the transition of oocyte to embryo, activation of MPK-1 (homology of ERK in mammals) phosphorylates Dcr-1 on two conserved Ser residues: S1705 (in RNase IIIb domain) and S1833 (in dsRBD domain), which is necessary and sufficient to trigger Dcr-1's nuclear translocation in *C. elegans*. This cellular translocation of Dcr-1 is also conserved in vertebrates. But phosphorylation of S1705 affects Dicer1 dicing activity, leading to accumulation of “pseudo-miRNAs,” carrying 2-5nt extensions on either 5′ or 3′ end of miRNAs [52].

## 6. Conclusion

Metabolic adaptation is vital for organismal survival. Despite those well recognized energy sensors including AMPK, mTOR, or autophagic process, Dicer and its products have an important role in modulating cellular adaptation to fluctuation in nutrient status. It seems that by producing a range of miRNAs, Dicer favors catabolism of fat, glucose and amino acids both in the CNS and some peripheral cells or tissues. With the increased epidemic rates of diseases associated with irregular energy metabolism, it is quite tempting that specific pharmacological compounds can be developed to target Dicer to affect its downstream miRNAs in the control of relevant metabolic diseases. But some questions still lay in the way: (1) involvements of Dicer in nutrient catabolism rely mostly on diverse miRNAs being produced. But the relationship between these metabolic-sensitive miRNAs, or whether the diversity of these miRNAs is due to cell/tissue-specificity, is far from being integrated; (2) despite its abundance of mRNA or protein level, modifications of Dicer's structure and cellular location also affect its miRNA producing activity. Nevertheless, it is assumed that homeostatic energy dysregulation could be restored by targeting Dicer with the researches deepening.
